# Bridging the Knowledge–Practice Gap: The Culturally Mediated Role of Attitude in Food Safety Behaviors During Pregnancy

**DOI:** 10.3390/foods14203564

**Published:** 2025-10-20

**Authors:** Hala Ayman Alyousef, Maria Alhadad, Tahani Ahmad Joukhadar, Nianhong Yang

**Affiliations:** 1 Hubei Key Laboratory of Food Nutrition and Safety, Department of Nutrition and Food Hygiene, School of Public Health, Tongji Medical College, Huazhong University of Science and Technology, Wuhan 430030, China; hala-al-yousef@hotmail.com; 2 Department of Nutrition, Health Science Faculty, Homs University, Homs 2841, Syriatahanijoukhadar99@gmail.com (T.A.J.)

**Keywords:** food safety, pregnant women, knowledge-attitude-practice, high-risk foods, structural equation modeling

## Abstract

Foodborne diseases pose serious risks during pregnancy; however, cross-cultural studies on their cognitive and behavioral factors that influence safe practices are lacking. This study aimed to compare the food safety knowledge, attitudes, and practices (KAPs) of pregnant women in China and Syria and to develop a model that explores how food safety knowledge (FSK) and attitudes (FSAs) influence different practices. A cross-sectional study was conducted among 808 Chinese and 815 Syrian pregnant women using a validated questionnaire. Data were analyzed using descriptive statistics, Chi-square, and Partial Least Squares Structural Equation Modeling. Chinese women demonstrated higher FSK and more positive FSAs (59.0% vs. 48.5%, *p* = 0.001) than Syrian women, whereas Syrian women showed stronger personal hygiene practices (mean score: 4.06 ± 0.68 vs. 3.93 ± 0.67, *p* = 0.001). While FSK directly influenced FSAs in both cohorts (China: β = 0.379, *p* < 0.001; Syria: β = 0.405, *p* < 0.001), its translation into practices was culturally specific. For Chinese women, FSA fully mediated the relationship between FSK and temperature control (TC) practices (indirect effect: β = 0.121, *p* < 0.001) and partially mediated personal hygiene and cross-contamination (CC) prevention. In contrast, for Syrian women, FSA mediated the relationship with high-risk food (HRF) avoidance (indirect effect: β = 0.092, *p* < 0.05) and personal hygiene (indirect β = 0.076, *p* < 0.05). The findings conclude that the pathways from knowledge to practice are complex and culturally mediated, indicating that effective public health interventions must be tailored to specific socio-cultural contexts to improve food safety behaviors.

## 1. Introduction

Pregnant women are particularly vulnerable to foodborne diseases (FBDs) because hormonal changes during pregnancy can weaken the immune system, increasing the risk of infections from various foodborne pathogens (FBPs) such as Listeria monocytogenes (*L. monocytogenes*), Toxoplasma gondii (*T. gondii*), Salmonella species, and Campylobacter jejuni [1,2]. Infections may lead to severe complications, including miscarriage, premature delivery, neonatal death, and neurological disorders in newborns, posing risks to both the pregnant woman and her unborn child [3,4]. For instance, congenital toxoplasmosis is one of the most common congenital infections in the world [5], with seroprevalence ranging from 15.2 to 28.5% among Chinese women [6,7,8,9,10] and around 26.7% among female university students in Syria [11], indicating significant exposure risk. Similarly, *L. monocytogenes* poses a high mortality risk for pregnant women in China, where approximately 26% of cases result in fatality, and 33–46% of infections lead to abortions, neonatal death, or fetal loss [12,13]. Because these life-threatening pathogens can be avoided by adhering to simple food safety practices (FSPs) such as proper cooking, hygiene, and refrigeration [14,15], improving food safety behaviors during pregnancy is a critical public health priority.

Assessing the knowledge, attitudes, and practices (KAPs) related to food safety among pregnant women is crucial for improving maternal and fetal health. Unfortunately, international research indicated that pregnant women possess gaps in both food safety knowledge (FSK) and FSPs [16,17,18]. Common unsafe practices include improper hand washing, cross-contamination (CC), and inadequate temperature control (TC) [19,20,21]. However, much of the existing literature in this domain involves descriptive or single-country studies that, while valuable for establishing baseline data, offer limited insight into the underlying cognitive and behavioral mechanisms [20,22]. Furthermore, while cultural context is recognized as important [23], few studies have employed a comparative quantitative design to empirically test and compare the pathways from knowledge to practice across distinct socio-cultural settings.

Although FSK and food safety attitude (FSA) are identified as critical cognitive factors influencing FSPs [24,25,26], their relationship with actual practices remains inconsistent. Many studies based on the KAP framework have shown that knowledge or attitudes do not always translate into safer food handling behaviors [27,28,29,30,31]. This inconsistency suggests that additional factors mediate and moderate this relationship, such as cultural norms, socioeconomic conditions, and information from healthcare providers (HCPs) [32,33,34,35]. To better understand these complex relationships, analytical models such as Partial Least Squares Structural Equation Modeling (PLS-SEM) provide an appropriate methodological approach to investigate both direct and indirect effects among variables while controlling for such potential confounders [36,37,38].

Yet, few studies have applied this approach in the context of food safety during pregnancy. Furthermore, there is a lack of research examining how specific types of practices, such as personal hygiene, CC prevention, TC, and high-risk food (HRF) avoidance, are differentially influenced by FSK and FSA. This study addresses these critical gaps and advances food safety research in three ways. First, it provides the first cross-cultural empirical comparison of food safety KAPs among pregnant women in China and Syria, two countries with markedly different cultural, economic, and health system contexts. Second, it extends the traditional KAP framework by explicitly modeling the mediating role of attitude in multiple domains of food safety behavior, helping to explain why FSK does not consistently result in safe practices. Third, by employing PLS-SEM, it offers a theoretically grounded and methodologically rigorous approach that quantifies the pathways through which FSK and FSA influence FSPs, uncovering culturally specific mechanisms that conventional analyses may overlook.

Understanding these culturally mediated pathways is vital for developing targeted, context-sensitive public health interventions that protect maternal and child health [39]. Accordingly, this study aims to (1) compare the KAPs related to food safety among pregnant women in China and Syria; (2) test the direct impact of FSK on FSAs; (3) explore the influence of FSAs on various FSPs, including TC, personal hygiene, prevention of CC, and HRF avoidance; and (4) assess the mediating role of FSAs in the relationship between FSK and these different practices. The research contributes new empirical evidence and a refined theoretical understanding of how culture shapes the translation of knowledge into food safety behavior. Additionally, this study aligns with the goals outlined in the “Outline of the Plan for Health China 2030,” which emphasizes the importance of health promotion education and spreading health-related scientific knowledge to ensure public health and well-being.

## 2. Theory and Hypotheses Development

The KAP model is one of the most widely used theories to explain and study FSPs [31]. This study used the KAP model as the theoretical foundation for establishing hypothesized relationships.

Previous studies have shown the importance of FSK in shaping FSPs [40,41]. Knowledge represents the information, understanding, and skills acquired through education or experience [42]. Numerous studies have also highlighted a significant correlation between FSK and FSAs [43,44], with a positive attitude serving as a key motivator for applying FSPs effectively [45]. According to the theory of planned behavior (TPB), individuals’ behavior is influenced by their intentions, and these intentions are shaped by their attitudes. Studies utilizing TPB have consistently shown that attitudes significantly influence individuals to perform safe food handling practices [46]. While knowledge is necessary for implementing FSPs [40,41], it does not automatically translate into behavior [30]. Individuals may have the knowledge of food safety principles but lack the motivation to implement them. Conversely, they may have a positive FSA but lack specific knowledge on how to implement safe practices effectively [47]. In this context, a positive FSA can serve as an intermediate factor that links knowledge to actual behavior. In particular, it is anticipated that positive FSAs will drive pregnant women to engage in FSPs such as TC, personal hygiene, preventing CC, and avoiding high-risk foods (HRFs).

**Figure 1:**
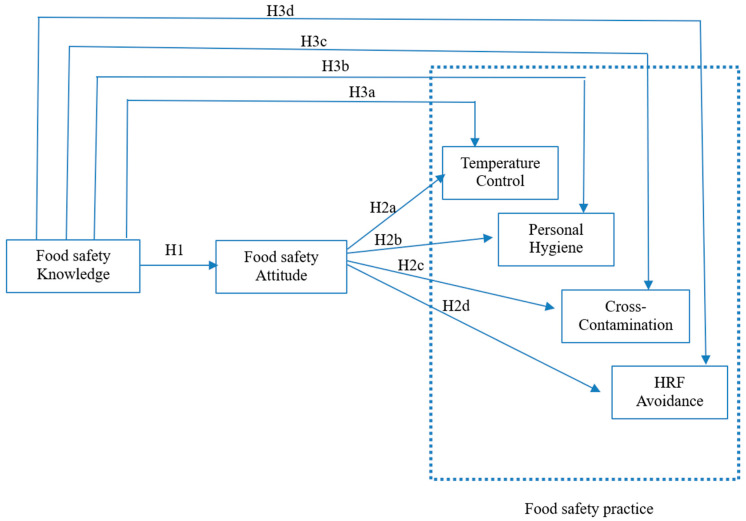
The research framework.

Based on the literature, we hypothesize (Figure 1):

**H1.**  *Pregnant women’s FSK has a direct positive impact on their FSAs.*

**H2.**  *Pregnant women’s FSAs positively influence their (a) TC practices, (b) personal hygiene practices, (c) CC practices, and (d) HRF avoidance.*

**H3.**  *The FSAs of pregnant women mediate the relationship between their FSK and the practice of (a) TC, (b) personal hygiene, (c) prevention of CC, and (d) HRF avoidance.*

## 3. Participants and Methods

### 3.1. Setting and Study Population

This cross-sectional study included pregnant women aged 18–45 years old in their third trimester who consented to participate in the study and held Syrian or Chinese nationality. The third trimester was selected due to heightened fetal vulnerability [48,49,50] and frequent prenatal visits. In Syria, participants were enrolled at Damascus Maternity Teaching Hospital (>11,000 annual deliveries [51]) and Maternity Children Hospital in Latakia (coastal city) to capture regional diversity. In China, the study was conducted in Tongji Hospital and Wuhan Children and Maternity Hospital in Wuhan, representing urban standardization.

### 3.2. Sample Collection Procedure

The study was conducted from August 2022 to February 2023. Pregnant women were invited to participate through a convenience sampling method. Participants completed a hard copy questionnaire, which was distributed and collected on the spot. Informed consent was obtained, and no incentives were offered for participation. An information sheet explaining the study’s aims was presented on the first page of the questionnaires. Research assistants were present to address any questions from the respondents. To reduce social desirability bias, questionnaires were anonymized, and participants were assured of confidentiality. As this study employed a convenience sampling method limited to pregnant women attending urban hospitals, the findings may not fully represent all pregnant women in either country, particularly those in rural settings. This design was chosen due to accessibility constraints and to ensure standardized data collection within comparable clinical environments. The potential implications of this sampling approach are further discussed in the limitations section.

### 3.3. Sample Size

The study’s minimum sample size was determined using Cochran’s Formula, considering an estimated population proportion of 50%, a margin of error of 0.05, and a critical z-value of 1.96 for a 95% confidence level. Based on these assumptions, an estimated minimum sample size of approximately 385 participants was required. A total of 1700 pregnant women were invited to participate in the study, with 850 from China and 850 from Syria.

### 3.4. Questionnaire Development Process

A structured questionnaire was developed through a methodical process including international guidelines, cultural adaptation, and rigorous validation.

#### 3.4.1. Questionnaire Design

An initial questionnaire was developed and refined based on a thorough review of relevant literature, utilizing validated scales and WHO recommendations on the five keys to safer food [21,52,53,54,55]. The questionnaire covered various domains related to food safety awareness. To ensure the questionnaire’s relevance and appropriateness, cultural factors specific to the target populations in both China and Syria were carefully considered. The questionnaire was written in English and then translated into Chinese and Arabic.

#### 3.4.2. Content Validity Assessment

Ten experts from the fields of nutrition, obstetrics, and public health (five Syrian, five Chinese) assessed content validity, focusing on cultural appropriateness and clarity. The Individual Content Validity Index (I-CVI) and Scale Content Validity Index/Average (S-CVI/Ave) were calculated [56]. All items lower than acceptable levels of content validity were deleted [56]. The final versions demonstrated high validity (Chinese: S-CVI/Ave = 0.90; Syrian Arabic: S-CVI/Ave = 0.92; Supplementary Table S1).

#### 3.4.3. Reliability Assessment

The reliability assessment aimed to ensure the consistency and stability of the questionnaire’s measurements, including two phases:

- Pilot Study Evaluation: This phase involved 50 Chinese pregnant women in Wuhan city and 50 Syrian pregnant women in Damascus city (not included in the study). Participant feedback indicated the clarity and comprehensibility of the questionnaire items, and no major revisions were considered necessary. Completion time fell within the expected range (from 10 to 15 min), and participants expressed overall willingness to participate. Data entry and coding procedures were efficient and accurate, with no major issues identified.
- Internal Consistency: Cronbach’s alpha coefficients for all KAP sections exceeded 0.70, indicating high internal consistency.
- Test–Retest Reliability: This phase aimed to evaluate response stability over time. Participants completed the questionnaire twice with a two-week interval to minimize recall bias. Test–retest reliability coefficients, such as Pearson’s correlation coefficient and Cohen’s Kappa, measured the agreement between responses across the two administrations. High test–retest reliability was observed, with strong correlations observed between responses at both times (Pearson’s r > 0.75; Cohen’s κ > 0.80; *p* < 0.001; Supplementary Table S2).

#### 3.4.4. Final Questionnaire Structure and Scoring

The final questionnaire included 5 sections (Supplementary Table S3).

Socio-demographic characteristics and sources of food safety information sections used multi-choice questions. The FSK section (8 items) aimed to evaluate pregnant women’s understanding of FBPs and safe food-handling practices. Participants rated statements on a 5-point agreement scale (1 = Strongly Disagree, 5 = Strongly Agree). For the reversed item (FSK6), the scale was inverted to ensure alignment with correct responses. Responses with a score of 4 or 5 were considered correct, while scores of 3 or below were considered incorrect. For the purpose of clear classification and to avoid the uncertainty of neutral responses, answers indicating clear agreement (“Agree” or “Strongly Agree”, corresponding to scores of 4 or 5) were classified as “correct.” A neutral response (score of 3) was considered to reflect a lack of confident knowledge and was thus categorized as “incorrect” for this binary assessment. This dichotomization is a common practice in KAP studies to distinguish those who are confident in their knowledge from those who are unsure or incorrect [57]. Total sum scores were also calculated for use in the PLS-SEM analysis, but for descriptive clarity and to align with public health intervention thresholds, total FSK scores were categorized as follows: Poor (≤24; ≤60%), Fair (25–31; >60%–<80%), or Good (≥32; ≥80%) [54,58].

FSAs (5 items) were measured on a 5-point Likert scale (1 = Strongly Disagree; 5 = Strongly Agree). Responses ≥4 indicated positive FSAs [59,60]. Total scores are defined as Negative for scores ≤ 15 (corresponding to ≤60%), Neutral for scores 16–19 (corresponding to >60%–<80%), or Positive for scores ≥20 (corresponding to ≥80%).

The FSPs section consisted of 16 items covering 4 key domains: personal hygiene (including 4 items), CC control (including 4 items), TC (including 3 items), and the consumption of HRFs during pregnancy (5 items). Behaviors were rated on a 5-point frequency scale ranging from Never to Always. For the HRF avoidance section, the scale was reversed. Practices scoring ≥4 were considered safe practices. The overall score was classified as Poor (≤48; ≤60%), Fair (49–63; >60%–<80%), or Good (≥64; ≥80%). The thresholds for these categories were selected based on established practices in KAP research [54,58], where a score of 60% or below is used to indicate an inadequate KAP level [54], and 80% or above indicates high proficiency [58]. Furthermore, because pregnant women are a highly vulnerable population with an increased risk of severe outcomes from FBDs, we adopted a stricter threshold. A score of ≤60% was therefore classified as “Poor” to reflect a low level of knowledge for this at-risk group, applying a heightened standard for assessing competency.

### 3.5. Data Analysis

Data analysis involved two stages: first, descriptive statistics summarized the data, while Chi-square tests measured correlations and independent *t*-tests compared the KAP components regarding food safety among pregnant women in China and Syria. The analysis was performed using IBM SPSS Statistics (version 23.0 IBM Corp., Armonk, NY, USA).

In the second stage, PLS-SEM was applied to assess direct and indirect relationships among KAP components. PLS-SEM was chosen due to its suitability for developing and testing theoretical models, offering advantages over the traditional covariance-based SEM, and it is particularly useful for exploratory research [60]. The analysis, performed using Smart-PLS 3.0 software, involved three steps: method bias evaluation, model assessment, and confirmatory factor analysis, ending with hypothesis testing [61].

#### 3.5.1. The Evaluation of Method Bias

The evaluation of method bias included assessing both non-response bias and common method bias (CMB). A *t*-test was employed, and the evaluation revealed no statistically significant differences between the means of variables in the first and last responses for both groups, indicating the absence of non-response bias. For CMB, full collinearity tests confirmed all VIFs < 3.3, supporting the absence of significant bias for both Chinese and Syrian data.

#### 3.5.2. Model Assessment and Factor Analysis

The analysis included two phases to evaluate the measurement model.

- Phase 1: reliability and scale assessment:

This phase evaluated the reliability and validity of the measurement model through a three-step analytical process. The results are presented in Table 1 for Chinese pregnant women and Table 2 for Syrian pregnant women.

First, the overall contribution of formative indicators to their respective constructs was examined by the indicators’ outer loadings. The results indicated that the outer loading of formative indicators for all constructs was above the threshold of 0.70, suggesting acceptable item reliability [62].

Second, internal reliability was assessed through composite reliability (CR). All constructs exhibited CR values above 0.7, exceeding the threshold for statistical significance [61]. Cronbach’s alpha was also used as another measure of internal reliability; Cronbach’s α exceeded 0.7 for all constructs [61]. Additionally, rho_A > 0.70 further validated reliability.

Third, convergent validity was verified by calculating the Average Variance Extracted (AVE) for each construct. The AVE values for each construct were all satisfactory, exceeding 0.5. This indicates that each construct explains at least 50% of the variance among its component items [63], providing further support for internal consistency and reliability of the measurement model.

- Phase 2: discriminant validity assessment:

Discriminant validity was assessed using three methodological approaches. The first method involved analyzing indicator cross-loadings to determine whether each indicator had the highest loading on its respective construct compared to other constructs in the model. This pattern was observed across both Chinese and Syrian participants (Table 3 for Chinese pregnant women and Table 4 for Syrian pregnant women).

Second, the AVE of each theoretical construct was compared to its squared inter-construct correlations, which indicates the shared variance between constructs in the path model. For discriminant validity, the AVE of each construct must exceed its squared correlation with any other construct in the model. All constructs satisfied this condition (Table 5), ensuring the construct explains more variance in its indicators than it shares with others [63].

Additionally, the heterotrait–monotrait (HTMT) ratio, shown in Table 5, was assessed as a final criterion for discriminant validity [64]. The HTMT ratio for all constructs met the recommended thresholds of below 0.85 for conceptually different constructs and 0.90 for conceptually similar constructs [60]. All methods confirmed discriminant validity for both cohorts.

#### 3.5.3. Testing Direct and Indirect Effect

The PLS approach estimates path coefficients and assesses the significance of these relationships using bootstrapping with a large number of subsamples (e.g., 5000). In this study, the T-statistics generated through bootstrapping were used to assess the significance of the relationships between the variables. Effects with |t| > 1.96 (*p* < 0.05) were considered significant. To ensure the robustness of the findings, this study accounted for age, education level, and access to information from HCPs, which could theoretically influence FSK, FSAs, and FSPs. By including these variables in the structural model, the analysis aimed to isolate their potential confounding effects and assess whether they affected the hypothesized relationships. The structural model’s explanatory power was assessed using the coefficient of determination (R^2^), which measures the amount of variance in each endogenous construct explained by its exogenous predictors [36,60].

## 4. Results

### 4.1. Demographic Characteristics and Food Safety Information Received

After excluding incomplete or inappropriate responses, the final analysis included 808 pregnant women from China and 815 pregnant women from Syria. The socio-demographic characteristics of these participants are detailed in Table 6. In both China and Syria, over half of pregnant women were aged ≤ 30 years, with no significant differences in age distribution between the two countries (*p* = 0.616). However, significant differences were observed in education levels between the two groups (*p* = 0.001).

Significantly more Chinese pregnant women (54.6%) reported receiving sufficient or plenty of food safety information during their pregnancies compared to Syrian women (44.0%) (*p* ˂ 0.001). While the internet was the most common source for both Chinese (42.0%) and Syrian pregnant women (47.3%), HCPs were considered the most trustworthy source of food safety information by most pregnant women, with a higher proportion among Syrian women (71.2%) compared to Chinese women (63.7%).

### 4.2. Food Safety KAPs of Pregnant Women

The FSK of Chinese and Syrian pregnant women is summarized in Table 7. The majority of Chinese women (77.8%) correctly recognized proper handwashing techniques (FSK4), whereas only 61.0% of Syrian women answered correctly (*p* < 0.001). Notably, both groups exhibited the lowest knowledge score regarding Listeria (FSK3). Only 33.5% of Chinese women and 29.8% of Syrian women identified the risk of Listeria infection, with no statistically significant difference between groups (*p* = 0.107).

When evaluating the overall level of FSK, Syrian women were more likely to demonstrate poor knowledge levels (43.8%) compared to Chinese women (38.6%) (*p* = 0.034). No significant difference was observed in the “good” knowledge level (*p* = 0.084).

Regarding FSAs, Chinese women reported greater interest in receiving food safety information (86.6% compared to 77.7%; *p* = 0.001) (Table 8) and stronger confidence in safe food choices (72.0% compared to 59.5%; *p* = 0.003). The assessment of FSAs shows that 59.0% of Chinese pregnant women held a positive FSA, which is significantly higher than the 48.5% observed among Syrian pregnant women (*p* = 0.001). Additionally, 23.8% of Syrian women exhibited a negative FSA level, compared to 9.4% of Chinese women (*p* = 0.001).

In FSPs, TC scored lowest for both groups (Chinese: 3.55 ± 0.76; Syrian: 3.56 ± 0.84; *p* = 0.859) (Table 9). Regarding personal hygiene, Syrian women achieved a significantly higher mean score (4.06 ± 0.68) than Chinese pregnant women (3.93 ± 0.67) (*p* = 0.001). However, this subscale showed the highest mean scores for both groups. In the CC subscale, Chinese pregnant women scored higher (3.80 ± 0.84) than Syrian women (3.67 ± 0.88) (*p* = 0.004). Furthermore, Chinese women exhibited significantly greater adherence to HRF avoidance (Chinese women: 3.92 ± 0.67; Syrian women: 3.76 ± 0.82; *p* < 0.001). Overall, 41.7% of Chinese pregnant women and 36.4% of Syrian pregnant women achieved a good level of FSP, while the highest percentage in both countries fell into the “fair” category.

### 4.3. Direct Relationships

**Figure 2:**
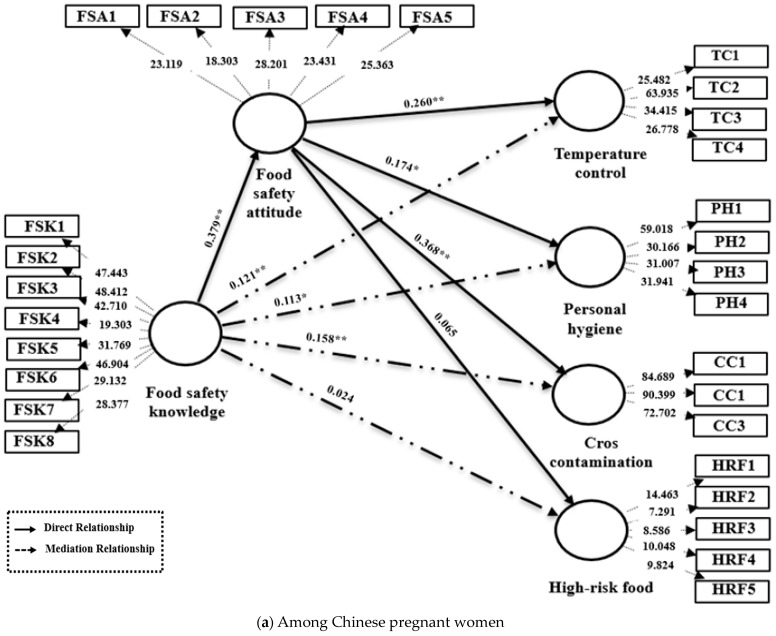

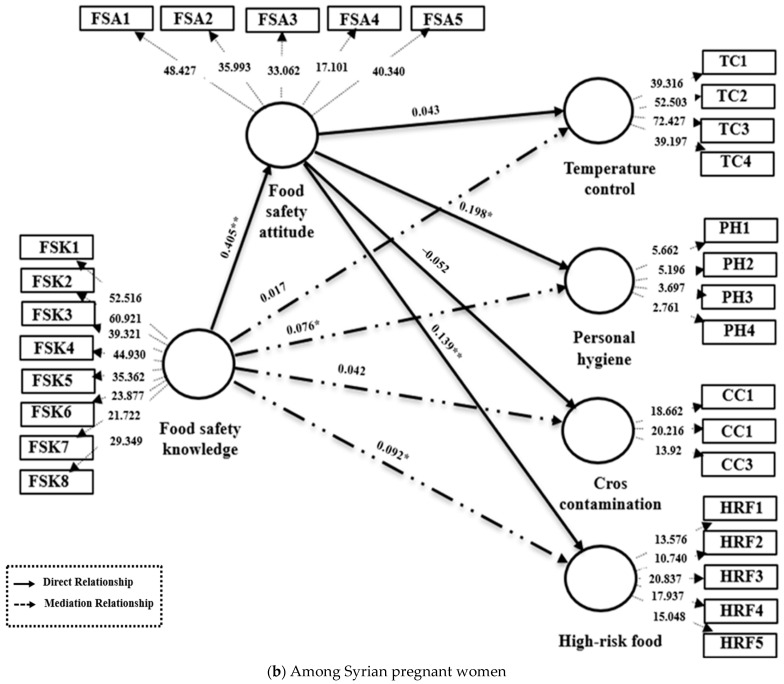
PLS-SEM model for (**a**) Chinese pregnant women and (**b**) Syrian pregnant women. This model shows the relationships between food safety knowledge, food safety attitudes, and food safety practice, highlighting both direct and mediating effects. Path coefficients (β) indicate the strength and direction of the relationships, with significant paths marked by asterisks (* *p* < 0.05, ** *p* < 0.01). Dotted arrows represent mediation relationships, and solid arrows represent direct relationships. Weights are provided for each indicator to show their contribution to the latent constructs.

The results of the direct relationship tests are presented in Table 10, with a visual representation provided in Figure 2.

Across both cohorts, the T-statistic for testing Hypothesis H1 was greater than the critical value of 1.96, indicating a significant positive relationship between FSK and FSA, thus strongly supporting H1 (Chinese: β = 0.379, *p* < 0.001; Syrian: β = 0.405, *p* < 0.001).

However, the influence of FSAs on specific practices differed between groups. Among Chinese pregnant women, FSA significantly improved TC (β = 0.260, *p* < 0.001), personal hygiene (β = 0.174, *p* < 0.005), and CC control (β = 0.368, *p* < 0.001), but did not affect HRF avoidance (*p* > 0.05). Conversely, Syrian women exhibited significant effects of FSA only on personal hygiene (β = 0.198, *p* < 0.05) and HRF avoidance (β = 0.139, *p* < 0.01), with non-significant relationships for TC and CC control. The structural model’s explanatory power was assessed using the coefficient of determination (R^2^). For Chinese participants, the model accounted for 38.2% of the variance in FSAs (R^2^ = 0.382) and between 26.5% and 42.7% of the variance in food safety practices (FSPs) across the four domains (R^2^_TC = 0.427, R^2^_personal hygiene = 0.312, R^2^_CC = 0.389, R^2^_HRF = 0.265). For Syrian participants, the model explained 41.0% of the variance in FSAs (R^2^ = 0.410) and 23.6–37.4% of the variance in FSPs (R^2^_TC = 0.236, R^2^_personal hygiene = 0.374, R^2^_CC = 0.281, R^2^_HRF = 0.267) [36,61]. The observed R^2^ values in this study indicate a weak-to-moderate level of explanatory power. This is acceptable and commonly observed in complex behavioral and cross-cultural research settings, where a significant portion of variance is often influenced by unmeasured contextual and sociological factors.

**Figure 3:**
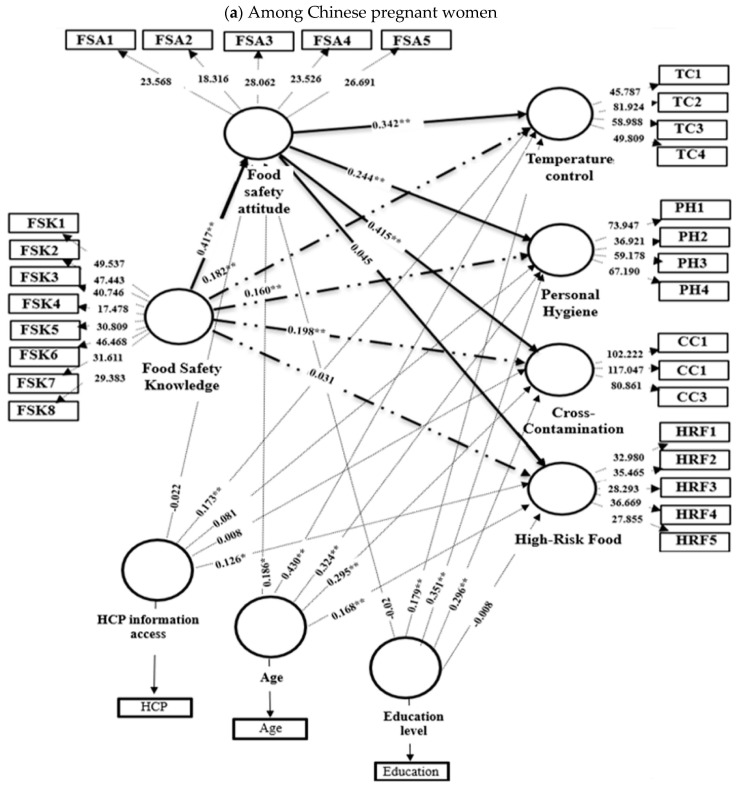

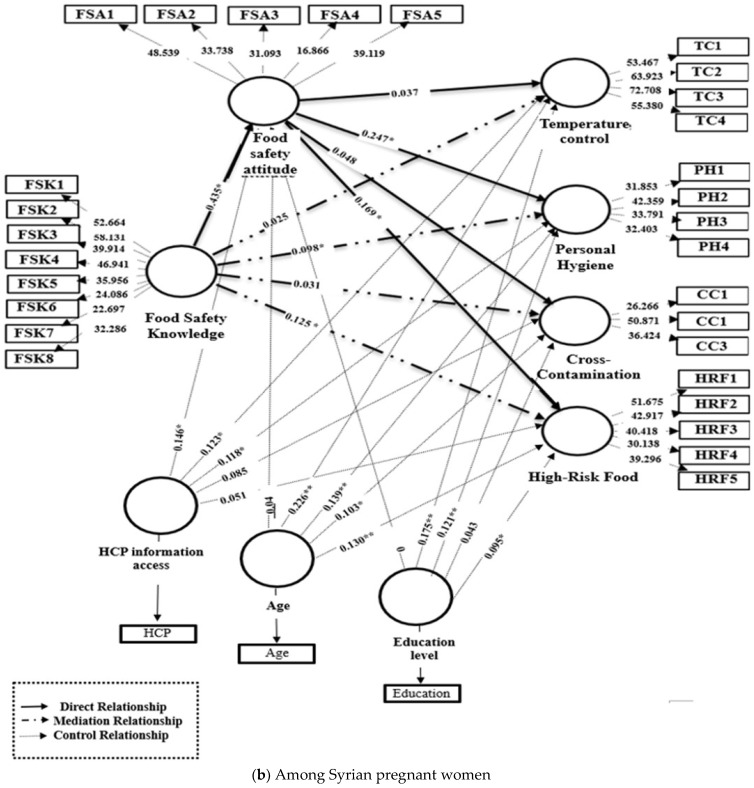
Extended PLS-SEM for (**a**) Chinese pregnant women and (**b**) Syrian pregnant women, including control variables (age, education level, HCP information access) for Chinese pregnant women. This model shows how control variables influence the relationships between food safety knowledge, food safety attitude, and food safety practice, highlighting both direct and mediating effects. Path coefficients (β) indicate the strength and direction of relationships, with significant paths marked by asterisks (* *p* < 0.05, ** *p* < 0.01). Dashed arrows represent control impacts, dotted arrows represent mediation relationships, and solid arrows represent direct relationships. Weights are provided for each indicator to show their contribution to the latent constructs.

To account for potentially confounding factors, the study included control variables in the analysis. The results of this extended model are presented in Table 11 for Chinese women and Table 12 for Syrian women, as well as shown in Figure 3. Even after controlling for age, education level, and access to HCP information, FSK showed a significant positive effect on FSAs in both Chinese (β = 0.417, *p* < 0.001) and Syrian cohorts (β = 0.435, *p* < 0.007). Furthermore, the influence of FSAs on specific practices remained consistent with baseline findings. Among Chinese pregnant women, FSA significantly predicted TC (β = 0.342, *p* < 0.001), personal hygiene (β = 0.244, *p* < 0.001), and CC control (β = 0.415, *p* < 0.001) but not HRF avoidance (*p* = 0.152). Conversely, among Syrians, FSA only significantly influenced personal hygiene (β = 0.247, *p* = 0.017) and HRF avoidance (β = 0.169, *p* = 0.007), with no significant effects on TC or CC control.

### 4.4. Mediation Relationships

Mediation analysis, as shown in Table 13 and Figure 2, revealed distinct cross-cultural patterns in attitude’s mediating role. For TC, Chinese women demonstrated a significant total effect of FSK on TC practices (β = 0.177, *p* < 0.001). When accounting for FSA, the direct effect became non-significant (*p* > 0.05), while the indirect effect through FSA remained significant (β = 0.121, *p* < 0.001), indicating full mediation with 68.3% of the total effect mediated. Conversely, Syrians exhibited no significant total, direct, or indirect effects.

Regarding personal hygiene, both groups demonstrated partial mediation. Chinese women had significant total (β = 0.354, *p* < 0.001), direct (β = 0.241, *p* < 0.05), and indirect effects (β = 0.113, *p* = 0.001). Syrian women also showed significant total (β = 0.315, *p* < 0.001), direct (β = 0.239, *p* < 0.001), and indirect effects (β = 0.076, *p* < 0.05), indicating that FSA partially mediates the relationship between FSK and personal hygiene among both groups.

For CC control, Chinese women displayed partial mediation (total: β = 0.436, *p* < 0.001; direct: β = 0.278, *p* < 0.001; indirect: β = 0.158, *p* < 0.001), while Syrians showed no mediation despite significant total (β = 0.193, *p* < 0.001) and direct effects (β = 0.151, *p* < 0.05), with non-significant indirect effects. Finally, for HRF avoidance, Chinese women exhibited no mediation (significant total: β = 0.253, *p* < 0.001; significant direct: β = 0.229, *p* = 0.05; non-significant indirect), whereas Syrians demonstrated partial mediation (significant total: β = 0.293, *p* < 0.001; direct: β = 0.201, *p* < 0.05; indirect: β = 0.092, *p* < 0.05).

To assess robustness, mediation analyses were repeated, controlling for age, education level, and HCP information access (Figure 3). Among Chinese pregnant women, the mediating role of FSA remained highly significant and robust for TC (β = 0.182, *p* < 0.001), personal hygiene (β = 0.160, *p* < 0.001), and CC control (β = 0.198, *p* < 0.001), confirming attitude’s consistent influence on translating knowledge into these practices. However, the non-significant mediation for HRF avoidance persisted (*p* = 0.647). For Syrian pregnant women, FSA mediation remained significant for personal hygiene (β = 0.098, *p* = 0.018) and emerged as significant for HRF avoidance after controls (β = 0.125, *p* = 0.008), demonstrating its robust role in these specific pathways. Mediation paths for TC and CC control in the Syrian group remained non-significant.

To provide a comprehensive overview of the key cross-cultural differences identified in this study, Table 14 summarizes the comparative patterns in KAP components and the culturally distinct direct and mediation pathways between Chinese and Syrian pregnant women. This synthesis highlights how the translation of knowledge into practice is mediated differently across cultural contexts.

## 5. Discussion

The findings of the present study highlight critical gaps in FSK among pregnant women in both China and Syria, raising concerns about their vulnerability to FBDs. This study identified a moderate level of FSK among Chinese and Syrian pregnant women, which is consistent with previous research indicating a widespread issue of insufficient FSK among pregnant women [17,18,21]. Similar studies conducted in multiple Chinese cities have reported inadequate FSK among the general public [65,66,67]. A particularly alarming finding was the insufficient knowledge regarding *Listeria*. This gap may be due to a lack of emphasis on *Listeria* in public health communications in both countries. Previous research has shown similar deficiencies in other populations; for example, only 12.2% of Portuguese pregnant women were aware of *Listeria* [53]. In contrast, pregnant women in Louisiana exhibited higher awareness, with the majority reporting knowledge of *Listeria* [21]. Understanding and assessing FSAs among pregnant women is critical for promoting and maintaining healthy practices. The positive attitudes among the majority of Chinese pregnant women likely reflect increased awareness and emphasis on hygiene and safety measures, particularly in response to the increased concerns during the COVID-19 pandemic [68]. Most Chinese participants reported paying more attention to food safety after the pandemic, indicating a heightened awareness shaped by intensive public messaging and the national “Healthy China 2030” campaign. This positive shift in attitude aligns with the widespread attention given to food safety during the pandemic, highlighting its impact on individual behaviors and practices [17,18,68]. In contrast, Syrian pregnant women exhibited a less optimistic attitude, which may be linked to challenges such as limited access to reliable information and socioeconomic limitations that constrain their ability to adopt and maintain FSPs, potentially leading to increased susceptibility to FBDs. Differences in healthcare delivery further contribute to these patterns. Chinese antenatal care systems are highly institutionalized and digitally networked, whereas Syrian systems rely more on face-to-face interactions in hospitals with limited educational resources. Consequently, the higher FSK and FSAs observed among Chinese participants likely reflect greater exposure to structured prenatal education and national health communication. Understanding how FSK influences FSAs and FSPs is crucial, especially for vulnerable groups such as pregnant women. Consistent with previous studies [43,44], FSK significantly influenced FSAs in both groups, confirming that interventions designed to enhance FSK could be an effective strategy for promoting more positive FSAs.

The examination of FSPs among pregnant women in China and Syria revealed significant areas of concern. Inadequate food handling practices at home significantly contribute to FBDs [69]. TC emerged as a critical aspect of food safety among both Chinese and Syrian women. This common challenge reflects a global issue, where pregnant women often lack specific knowledge regarding safe TC practices for food preparation and storage [19,20]. However, the culturally different patterns in how FSAs translate into behavior highlight that knowledge and positive attitudes alone do not consistently produce safer practices. In China, attitudes appear to be a key motivational factor linking FSK to TC. This may be because proper TC often requires intentional effort and access to reliable appliances, which are more common in urban Chinese households. These findings are in line with a previous study, which indicated that FSAs mediate the relationship between knowledge and behavior [30]. In contrast, the dynamics are different for Syrian pregnant women. These variations in the role of FSAs between the two countries suggest that cultural or contextual factors may differently shape the relationship between FSK, FSA, and TC practices for Chinese and Syrian populations. The strong mediating role of FSAs on personal hygiene in both cohorts contrasts with previous literature, which did not observe a direct or mediation role of FSAs on personal hygiene practices [47]. This difference could be attributed to the unique context of our study, which was conducted during the COVID-19 pandemic. Research conducted specifically during the pandemic has reported a significant global shift in hygiene awareness. For instance, studies from the United States and Jordan reported that the pandemic profoundly heightened public attention to handwashing and sanitation, although often motivated by virus avoidance rather than traditional food safety concerns [17,18,68,70]. In China, extensive public messaging and digital surveillance during the pandemic reinforced daily hygiene and food-handling vigilance. Similarly, in Syria, pandemic-related disruptions to healthcare and public communication were more pronounced. This may help explain why the attitude–behavior link for hygiene was stronger post-pandemic than in the earlier literature [47]. CC control practices also varied culturally. The positive direct and mediation role of FSAs observed only among Chinese women aligns with the broader trend in the food safety literature, where approximately half of the conducted studies related to food handling, knowledge, and/or attitude do not consistently lead to improved practices [30,31]. This inconsistency may be attributed to the complexity of translating knowledge into practice, which is influenced by multiple factors that mediate and moderate this relationship [32,34]. Structured prenatal programs in major Chinese cities like Wuhan routinely integrate hygiene counseling, and widespread smartphone use enables continuous access to official health information. These structural and informational supports may strengthen the translation of knowledge into practice, particularly for procedural behaviors such as CC prevention, where skills and resources are needed. In contrast, Syrian pregnant women face resource limitations, ongoing economic difficulty, and infrastructural instability, including irregular electricity and refrigeration access, which constrain the ability to apply knowledge of CC control.

While good nutrition is strongly recommended during pregnancy, avoiding HRFs is important [71]. However, this study found a notable trend of HRF consumption among both Chinese and Syrian pregnant women. This aligns with prior research indicating that pregnant women continue to consume HRFs despite the elevated risks of FBDs [17,18,21].

The relationship between FSAs and HRF practices showed different patterns and underscores the complex nature of factors shaping HRF intake behaviors in these countries. The significant relationship between FSAs and HRF intake practices was observed only among Syrian participants. This can be understood through dietary culture. In Syria, dishes like raw meat-based Kibbeh are traditional and culturally significant. Avoiding such foods requires not just knowledge of the risk but a strong attitude that prioritizes safety over deep-seated food traditions. This attitude is less critical for practices like personal hygiene, which may be more habitual. Among Chinese women, the absence of a significant FSK or FSA effect on HRF avoidance suggests that other determinants, such as convenience culture, online food delivery, or trust in commercial food systems, may influence behavior. These variations emphasize that the knowledge–behavior relationship is context-specific, and it aligns with broader trends in the food safety literature, where the translation of knowledge into improved practices is not universally evident [30,31]. This confirmed the complexity of the knowledge–behavior relationship, emphasizing the necessity for complicated interventions that extend beyond information provision alone.

## 6. Conclusions

This study offers a novel comparative insight into food safety KAPs among urban pregnant women in China and Syria, addressing critical gaps in maternal health research. The analysis reveals significant cross-country differences: Chinese women demonstrated higher FSK and more positive FSAs, while Syrian women exhibited stronger adherence to personal hygiene practices. Using PLS-SEM, this research examined the complex relationships between KAP components. The results indicate that FSK significantly enhanced FSAs in both populations, with attitudes serving as a crucial mediator between FSK and specific practices. However, the mediation patterns revealed notable cultural variations. For Chinese women, FSA fully mediated the relationship between FSK and TC practices and partially mediated both personal hygiene and CC prevention. In contrast, among Syrian women, FSAs mediated only the FSK and HRF relationship. These findings demonstrate that the pathways from knowledge to practice are culturally specific rather than universal. Consequently, public health interventions aimed at improving food safety during pregnancy need culturally tailored approaches that improve women’s awareness. Future research should focus on identifying the underlying cultural factors behind these differences to develop more effective and targeted educational programs that address the specific behavioral and attitudinal factors relevant to each socio-cultural context.

### 6.1. Practical, Theoretical, and Social Implications

The findings of this study carry significant implications.

#### 6.1.1. Practical Implications

Practically, the findings provide clear, actionable guidance for public health authorities and healthcare providers to develop targeted, culturally sensitive educational programs. In China, where knowledge and attitudes are relatively strong, but gaps persist in TC and CC prevention, prenatal education modules should emphasize hands-on demonstrations and visual materials showing correct refrigeration, reheating, and separation techniques. Given the high internet usage, create official, hospital-authorized content for social media platforms (e.g., WeChat) and pregnancy apps. This content should focus on building positive attitudes by emphasizing the direct mother–baby health benefits of these safe practices. In Syria, where knowledge and attitudes are weaker, but personal hygiene practices are stronger, interventions should focus on translating knowledge into sustained behavior by addressing structural and motivational barriers. For example, develop simple, attractive leaflets and posters for clinic waiting rooms. The messaging should be direct and solution-oriented, focusing on how to implement practices. For example, visuals could show the “right way” to wash hands and provide a definitive list of local high-risk foods to avoid.

As HCPs were identified as the most trusted source of information in both countries, prenatal care visits are a critical intervention point. Structured food safety counseling should be inserted into routine prenatal care. A standardized, 10–15 min “food safety module” could be included during antenatal visits, covering key messages aligned with the WHO “Five Keys to Safer Food.” Training programs for obstetricians, nurses, and nutrition educators should include communication skill-building to deliver culturally relevant and non-technical messages. Partnering with local health authorities can ensure that educational content reflects cultural food habits (e.g., traditional dishes, seasonal foods).

#### 6.1.2. Theoretical Implications

Theoretically, the study empirically demonstrates that the KAP model is not a one-size-fits-all framework. The varying mediating strength of FSA between the two cohorts provides strong evidence that the model’s pathways are moderated by external cultural and socioeconomic factors and provides a nuanced theoretical explanation for why the knowledge–practice gap varies across behaviors and cultural contexts. This study directly addresses the identified gap in the literature concerning KAP model extensions [39], as it is the first to empirically explore and model the specific mechanisms linking knowledge to specific practices like TC, personal hygiene, HRFs, and CC prevention. This research advances behavioral theory by validating a more complex KAP framework and provides a replicable methodology for future research.

#### 6.1.3. Social Implications

Socially, this study aligns directly with initiatives like China’s “Healthy China 2030” plan. By offering an evidence-based strategy to reduce FBDs, the study contributes to improving maternal and child health outcomes across different contexts.

### 6.2. Limitations and Future Research

Despite its contributions, this study has some limitations. Its cross-sectional design prevents causal interpretations of the relationships between KAP variables; while our model tests hypothesized pathways, the data cannot confirm causality. Future longitudinal or intervention studies are needed to assess causal relationships and observe how KAPs change over time.

The geographic focus on urban pregnant women in Wuhan, Damascus, and Latakia may affect the generalizability of the findings. Wuhan is a major, well-developed urban center with an advanced healthcare system. The findings here may not fully represent the experiences of pregnant women in peri-urban or rural areas of China, where access to information and healthcare resources may differ. Similarly, while Damascus and Latakia provide regional diversity within Syria, the rural populations may face distinct food safety challenges (e.g., limited access to healthcare). Therefore, the results may not be generalizable to other Syrian populations. Future studies should expand sampling to rural areas and other regions. The use of convenience sampling may introduce a selection bias, as participants were recruited based on accessibility and willingness to participate. This limits the representativeness of the sample and may reduce the generalizability of the findings to broader populations. Future studies could address these limitations by employing probability sampling methods (e.g., random or stratified sampling) to enhance representativeness.

Furthermore, the reliance on self-reported practices may be influenced by social bias. The potential for social desirability bias is a particular concern, which may be reflected in the very high self-reported scores for personal hygiene practices in both cohorts. Participants may have over-reported adherence to socially desirable behaviors like handwashing, meaning the actual compliance might be lower. Recall bias is also a potential concern, as participants’ reporting of their dietary habits and practices may not be fully accurate. Observational or mixed-method approaches could be added to future studies to validate self-reported behaviors and provide richer behavioral insights.

Finally, data collection during the pandemic may have uniquely heightened hygiene awareness among participants, potentially limiting direct comparability with pre- or post-pandemic conditions. Future studies across diverse timeframes are needed to clarify long-term trends.

## Figures and Tables

**Table 1:** Measurement model for Chinese pregnant women’s data.

	Items	Loadings	AVE	CR	Rho_A	Cronbach’s Alpha
FSK	FSK1	0.803	0.55	0.906	0.895	0.882
	FSK2	0.802				
	FSK3	0.773				
	FSK4	0.786				
	FSK5	0.74				
	FSK6	0.769				
	FSK7	0.715				
	FSK8	0.719				
FSA	FSA1	0.718	0.511	0.839	0.766	0.761
	FSA2	0.759				
	FSA3	0.743				
	FSA4	0.712				
	FSA5	0.737				
TC	TC1	0.788	0.672	0.891	0.883	0.839
	TC2	0.886				
	TC3	0.815				
	TC4	0.786				
Personal hygiene	PH1	0.875	0.698	0.902	0.882	0.857
	PH2	0.816				
	PH3	0.812				
	PH4	0.837				
CC	CC1	0.898	0.804	0.925	0.88	0.878
	CC2	0.909				
	CC3	0.883				
HRFs avoidance	HRF1	0.869	0.568	0.867	0.923	0.829
	HRF2	0.703				
	HRF3	0.721				
	HRF4	0.731				
	HRF5	0.733				

All Item Loadings > 0.7 indicates Indicator Reliability [62]. All Average Variance Extracted (AVE) > 0.5 indicates Convergent Reliability [63]. All composite reliability (CR) > 0.7 indicates internal consistency [60]. All Cronbach’s alpha > 0.7 indicates Indicator Reliability [64].

**Table 2:** Measurement model for Syrian pregnant women.

	Items	Loadings	AVE	CR	Rho_A	Cronbach’s Alpha
FSK	FSK1	0.811	0.561	0.91	0.892	0.887
	FSK2	0.824				
	FSK3	0.763				
	FSK4	0.798				
	FSK5	0.753				
	FSK6	0.711				
	FSK7	0.702				
	FSK8	0.721				
FSA	FSA1	0.821	0.589	0.877	0.851	0.828
	FSA2	0.789				
	FSA3	0.775				
	FSA4	0.727				
	FSA5	0.774				
TC	TC1	0.81	0.71	0.907	0.898	0.865
	TC2	0.856				
	TC3	0.889				
	TC4	0.813				
Personal hygiene	PH1	0.875	0.586	0.847	0.879	0.795
	PH2	0.862				
	PH3	0.719				
	PH4	0.731				
CC	CC1	0.843	0.706	0.878	0.824	0.797
	CC2	0.874				
	CC3	0.801				
HRF avoidance	HRF1	0.843	0.652	0.903	0.908	0.872
	HRF2	0.775				
	HRF3	0.841				
	HRF4	0.818				
	HRF5	0.758				

**Table 3:** Indicator items cross-loading among Chinese pregnant women.

	FSK	FSA	TC	PH	CC	HRF
FSK1	**0.803**	0.256	0.112	0.158	0.179	0.111
FSK2	**0.802**	0.245	0.052	0.092	0.165	0.137
FSK3	**0.773**	0.248	0.086	0.11	0.172	0.161
FSK4	**0.786**	0.244	0.029	0.003	0.134	0.135
FSK5	**0.74**	0.237	0.088	0.087	0.181	0.067
FSK6	**0.769**	0.349	0.169	0.208	0.269	0.07
FSK7	**0.715**	0.275	0.048	0.021	0.127	0.077
FSK8	**0.719**	0.272	0.122	0.128	0.186	0.113
FSA1	0.271	**0.718**	0.127	0.131	0.202	0.053
FSA2	0.278	**0.759**	0.073	0.031	0.111	0.117
FSA3	0.281	**0.743**	0.147	0.137	0.233	0.067
FSA4	0.223	**0.712**	0.165	0.131	0.234	0.083
FSA5	0.256	**0.737**	0.168	0.149	0.235	0.065
TC1	0.058	0.158	**0.788**	0.42	0.449	0.033
TC2	0.155	0.202	**0.886**	0.543	0.544	0.102
TC3	0.122	0.125	**0.815**	0.471	0.498	0.09
TC4	0.063	0.134	**0.786**	0.465	0.47	0.068
PH1	0.165	0.167	0.531	**0.875**	0.561	0.045
PH2	0.145	0.112	0.476	**0.816**	0.489	0.007
PH3	0.098	0.144	0.451	**0.812**	0.523	0.076
PH4	0.071	0.128	0.479	**0.837**	0.524	0.047
CC1	0.227	0.276	0.58	0.637	**0.898**	0.034
CC2	0.22	0.237	0.536	0.551	**0.909**	0.041
CC3	0.215	0.265	0.497	0.5	**0.883**	0.105
HRF1	0.155	0.14	0.12	0.114	0.117	**0.869**
HRF2	0.015	−0.014	0	−0.042	−0.071	**0.703**
HRF3	0.083	0.03	0.025	0.004	0.003	**0.721**
HRF4	0.092	0.031	0.035	−0.005	0.001	**0.731**
HRF5	0.085	0.068	0.061	−0.019	0.026	**0.733**

Note: Average Variance Extracted (AVE) values are in bold.

**Table 4:** Indicator items cross-loading among Syrian pregnant women.

	FSK	FSA	TC	PH	CC	HRF
FSK 1	**0.811**	0.309	0.222	0.111	0.132	0.113
FSK 2	**0.824**	0.311	0.179	0.069	0.106	0.06
FSK 3	**0.763**	0.255	0.163	0.058	0.111	0.054
FSK 4	**0.798**	0.302	0.135	0.028	0.067	0.055
FSK 5	**0.753**	0.325	0.146	0.076	0.063	0.043
FSK 6	**0.711**	0.328	0.122	0.009	0.05	0.024
FSK 7	**0.702**	0.257	0.096	0.055	0.073	0.015
FSK8	**0.721**	0.315	0.138	0.034	0.085	0.084
FSA1	0.367	**0.821**	0.162	0.108	0.035	−0.041
FSA2	0.377	**0.789**	0.111	−0.018	−0.041	−0.085
FSA3	0.28	**0.775**	0.081	0.047	0.028	−0.093
FSA4	0.172	**0.727**	0.091	0.056	−0.008	−0.079
FSA5	0.283	**0.774**	0.1	0.058	0.01	−0.022
TC1	0.137	0.112	**0.81**	0.092	0.033	−0.021
TC2	0.171	0.098	**0.856**	0.124	0.026	0.029
TC3	0.214	0.168	**0.889**	0.129	0.045	−0.006
TC4	0.15	0.099	**0.813**	0.114	0.049	−0.026
PH1	0.073	0.069	0.105	**0.875**	0.109	0.15
PH2	0.071	0.055	0.114	**0.862**	0.075	0.084
PH3	0.027	0.033	0.142	**0.719**	0.052	0.027
PH4	0.025	−0.012	0.083	**0.731**	0.045	0.1
CC1	0.11	−0.004	0.037	0.082	**0.843**	0.027
CC2	0.114	0.037	0.075	0.104	**0.874**	0.057
CC3	0.055	−0.035	−0.012	0.064	**0.801**	0.042
HRF1	0.083	−0.042	0.004	0.032	0.078	**0.843**
HRF2	0.032	−0.019	0.008	0.066	0.06	**0.775**
HRF3	0.102	−0.059	0.015	0.137	0.031	**0.841**
HRF4	0.024	−0.127	−0.034	0.128	0.038	**0.818**
HRF5	0.052	−0.034	−0.012	0.107	−0.005	**0.758**

Note: Average Variance Extracted (AVE) values are in bold.

**Table 5:** Discriminant validity for Chinese and Syrian cases: Fornell and Larcker criteria and HTMT.

	FSA	FSK	HRF	PH	TC	CC
a Fornell and Larcker criteria						
Chinese pregnant women						
FSA	0.715					
FSK	0.364	0.741				
HRFs	0.105	0.144	0.754			
Personal hygiene	0.168	0.15	0.052	0.835		
TC	0.195	0.128	0.092	0.583	0.82	
CC	0.29	0.246	0.067	0.629	0.601	0.897
Syrian pregnant women						
FSA	0.767					
FSK	0.402	0.749				
HRFs	−0.081	0.078	0.807			
Personal hygiene	0.064	0.075	0.124	0.766		
TC	0.146	0.204	−0.006	0.138	0.842	
CC	0.004	0.116	0.049	0.101	0.046	0.84
b HTMT						
Chinese pregnant women						
FSA	1					
FSK	0.441	1				
HRFs	0.106	0.141	1			
Personal hygiene	0.197	0.155	0.082	1		
TC	0.231	0.131	0.088	0.677	1	
CC	0.346	0.271	0.087	0.72	0.694	1
Syrian pregnant women						
FSA	1					
FSK	0.448	1				
HRFs	0.096	0.083	1			
Personal hygiene	0.09	0.088	0.139	1		
TC	0.162	0.223	0.031	0.169	1	
CC	0.053	0.13	0.064	0.108	0.061	1

a. The diagonal is the square root of the AVE of the latent variables and indicates the highest in any column or row [63]. b. For conceptually similar constructs: HTMT < 0.90. For conceptually different constructs: HTMT < 0.85 [60].

**Table 6:** Socio-demographic characteristics of Chinese and Syrian pregnant women.

Demographic Information	Chinese Pregnant Women	Syrian Pregnant Women	*p*
N	808	815	
Age n (%)			
≤30 years	459 (56.8)	473 (58.0)	0.616
>30 years	349 (43.2)	342 (42.0)	
Highest educational level n (%)			
High school or below	370 (45.8)	438 (53.7)	0.001
College or above	438 (54.2)	377 (46.3)	
First-time pregnancy n (%)			
No	409 (50.6)	486 (59.6)	0.001
Yes	399 (49.4)	329 (40.4)	
History of abortion n (%)			
No	489 (60.5)	530 (65.0)	0.060
Yes	319 (39.5)	285 (35.0)	
Planned pregnancy n (%)			
No	361 (44.7)	475 (58.3)	0.001
Yes	447 (55.3)	340 (41.7)	
Number of children n (%)			
0 children	468 (57.9)	367 (45.0)	0.001
≥1 child	340 (42.1)	448 (55.0)	
Current job n (%)			
No	271 (33.5)	444 (54.5)	0.001
Yes	537 (66.5)	371 (45.5)	
Amount of information received n (%)			
None or limited	367 (45.4)	456 (56.0)	˂0.001
Sufficient or plenty	441 (54.6)	359 (44.0)	
Sources of received information n (%)			
HCPs	257 (31.8)	286 (35.1)	0.161
Internet	339 (42.0)	386 (47.3)	0.028
Television and radio	153 (18.9)	87 (10.7)	˂0.001
Other	59 (7.3)	56 (6.9)	0.735
Most trustworthy source n (%)			
HCPs	515 (63.7)	580 (71.2)	0.001
Internet	154 (19.1)	169 (20.7)	0.398
Television and radio	113 (14.0)	49 (6.0)	˂0.001
Other	26 (3.2)	17 (2.1)	0.201

**Table 7:** Food safety knowledge among Chinese (n = 808) and Syrian (n = 815) pregnant women.

Item Code	Statement	Chinese Pregnant Women N (%)	Syrian Pregnant Women N (%)	*p*
Knowledge of FBPs ^a^				
FSK1	Salmonella	431 (53.3)	370 (45.4)	0.001
FSK2	Toxoplasma	333 (41.2)	300 (36.8)	0.069
FSK3	Listeria	271 (33.5)	243 (29.8)	0.107
Knowledge of safe food handling ^a^				
FSK4	Handwashing practices	629 (77.8)	497 (61.0)	0.001
FSK5	Refrigeration prevents foodborne illness	521 (64.5)	520 (63.8)	0.776
FSK6	Tasting to check food doneness	530 (65.6)	458 (56.2)	0.001
FSK7	Thawing frozen foods safely	510 (63.1)	557 (68.3)	0.027
FSK8	Food separation practices	533 (66.0)	623 (76.4)	0.001
Level of total food safety knowledge ^b^				
Poor		312 (38.6)	357 (43.8)	0.034
Fair		231 (28.6)	223 (27.4)	0.582
Good		265 (32.8)	235 (28.8)	0.084

Note: ^a^: N (%) Responses with a score of 4 or 5 were considered correct, indicating high or moderate confidence. ^b^: The percentages indicate the proportion of Chinese or Syrian pregnant women within each category.

**Table 8:** Descriptive statistics of food safety attitude among Chinese (n = 808) and Syrian (n = 815) pregnant women.

Item Code	Statement	Chinese Women N (%)	Syrian Women N (%)	*p*
FSA1	You are interested in receiving information about food safety during pregnancy.	700 (86.6)	633 (77.7)	0.001
FSA2	You have paid more attention to food safety after the COVID-19 pandemic.	719 (89.0)	620 (76.1)	0.001
FSA3	Your food safety behaviors have improved after the COVID-19 pandemic.	657 (81.3)	519 (63.7)	0.001
FSA4	You believe that having proper knowledge about food safety is important for the health of both you and your baby.	610 (75.5)	554 (68.0)	0.001
FSA5	You are confident in your ability to make safe food choices during pregnancy.	582 (72.0)	485 (59.5)	0.003
	Level of food safety attitude			
	Negative	76 (9.4)	194 (23.8)	0.001
	Neutral	255 (31.6)	226 (27.7)	0.091
	Positive	477 (59.0)	395 (48.5)	0.001

Note: N (%) represents the number and percentage of participants who reported a positive attitude (“agree” or “strongly agree”). The percentages indicate the proportion of Chinese or Syrian pregnant women within each category.

**Table 9:** Descriptive statistics of food safety practices among Chinese (n = 808) and Syrian (n = 815) pregnant women.

Item Code	Statement	Chinese Women N (%)	Syrian Women N (%)	*p*
Temperature control practices ^a^				
TC1	Refrigerate cooked food or leftovers within two hours of cooking.	481 (59.5)	451 (55.3)	0.088
TC2	Thoroughly reheating all cooked foods or leftovers to a boil before eating.	505 (62.5)	526 (64.5)	0.393
TC3	Keeping freezer at or below −18 °C (0 °F).	435 (53.8)	497 (61.0)	0.004
TC4	Ensuring that ready-to-eat food is steaming hot before eating.	458 (56.7)	405 (49.7)	0.005
	Mean ± SD	3.55 ± 0.76	3.56 ± 0.84	0.859
Personal hygiene practices ^a^				
PH1	Wash your hands well before eating.	599 (74.1)	695 (85.3)	0.001
PH2	Wash your hands well before starting to prepare or cook food.	673 (83.3)	690 (84.7)	0.452
PH3	Wash your hands well after touching raw egg.	637 (78.8)	590 (72.4)	0.003
PH4	Wash your hands well after touching raw meat or chicken.	702 (86.9)	724 (88.8)	0.228
	Mean ± SD	3.93 ± 0.67	4.06 ± 0.68	0.001
Cross-contamination prevention ^a^				
CC1	Rinse cutting boards, knives used for raw chicken or meat with hot water and soap before using them for other food.	608 (75.2)	561 (68.8)	0.004
CC2	Keep raw food separately from cooked food.	544 (67.3)	602 (73.9)	0.004
CC3	Use different chopsticks or spoons for uncooked ingredients (meat, poultry, or seafood) and cooked ingredients.	510 (63.1)	448 (55.0)	0.001
	Mean ± SD	3.80 ± 0.84	3.67 ± 0.88	0.004
High-risk food avoided during pregnancy ^a^				
HRF1	Pre-prepared salad or pre-cut fruits.	719 (89.0)	709 (87.0)	0.217
HRF2	Unwashed raw fruits or vegetables.	710 (87.9)	632 (77.5)	0.001
HRF3	Ready-to-eat meat served without being steaming hot.	526 (65.1)	442 (54.2)	0.001
HRF4	Undercooked meat or foods containing raw meat (e.g., hotpot, barbeque, raw kibbeh).	602 (74.5)	545 (66.9)	0.001
HRF5	Raw or undercooked eggs, pre-prepared (boiled or fried) egg stored at room temperature.	705 (87.3)	475 (58.3)	0.001
	Mean ± SD	3.92 ± 0.67	3.76 ± 0.82	0.001
	Level of total food safety knowledge ^b^			
	Poor	70 (8.7)	53 (6.6)	0.100
	Fair	401 (49.6)	465 (57)	0.002
	Good	337 (41.7)	297 (36.4)	0.029

Note: ^a^: N (%) represents the number and percentage of participants who reported safe practices. ^b^: The percentages indicate the proportion of Chinese or Syrian pregnant women within each category.

**Table 10:** Direct relationships hypothesis testing among Chinese and Syrian pregnant women.

Hypothesis	Std Beta	Std Error	*t*-Statistic	Decision	97.5%CI LL	97.5%CI UL
Among Chinese pregnant women						
H1: FSK → FSA	0.379 **	0.062	6.061	Supported	0.245	0.501
H2a: FSA → TC	0.260 **	0.048	5.357	Supported	0.163	0.358
H2b: FSA → personal hygiene	0.174 *	0.051	3.371	Supported	0.167	0.267
H2c: FSA → CC	0.368 **	0.033	11.055	Supported	0.30	0.427
H2d: FSA → HRF avoidance	0.065	0.052	0.815	Not supported	−0.089	0.146
Among Syrian pregnant women						
H1: FSK → FSA	0.405 **	0.029	13.804	Supported	0.348	0.464
H2a: FSA → TC	0.043	0.052	0.778	Not Supported	−0.09	0.129
H2b: FSA → personal hygiene	0.198 *	0.037	2.482	Supported	0.112	0.248
H2c: FSA → CC	−0.052	0.048	1.069	Not Supported	−0.141	0.036
H2d: FSA → HRF avoidance	0.139 **	0.037	3.581	Supported	0.098	0.205

** *p* < 0.001, * *p* < 0.05.

**Table 11:** Direct relationships hypothesis testing among Chinese pregnant women considering control variables.

Relationships	Std Beta	Std Error	*t*-Statistic	*p*-Value	Decision
FSK → FSA	0.417	0.037	10.67	<0.001	Supported
FSA → TC	0.342	0.032	7.444	<0.001	Supported
FSA → personal hygiene	0.244	0.031	6.363	<0.001	Supported
FSA → CC	0.415	0.032	8.834	<0.001	Supported
FSA → HRF	0.045	0.042	1.435	0.152	Not Supported
Age → FSA	0.186	0.04	2.197	0.028	-
Age → TC	0.430	0.033	12.875	<0.001	-
Age → personal_hygiene	0.324	0.03	10.9	<0.001	-
Age → CC	0.295	0.032	9.204	<0.001	-
Age → HRF	0.168	0.043	3.89	<0.001	-
Education level → FSA	−0.02	0.036	0.588	0.557	-
Education level → TC	0.179	0.035	5.191	<0.001	-
Education level → personal_hygiene	0.351	0.028	12.473	<0.001	-
Education Level → CC	0.296	0.03	9.912	<0.001	
Education Level → HRF	−0.008	0.039	0.189	0.85	-
HCP information access → FSA	−0.022	0.038	0.581	0.562	-
HCP information access → TC	0.173	0.034	5.18	<0.001	-
HCP information access → personal_hygiene	0.081	0.031	2.668	0.008	-
HCP information access → CC	−0.008	0.033	0.212	0.832	-
HCP information access → HRF	0.126	0.043	2.883	0.004	-

Note: A dash (-) in the Decision column indicates that the relationship is for a control variable and not a direct hypothesis being tested.

**Table 12:** Direct relationships hypothesis testing among Syrian pregnant women considering control variables.

Relationships	Std Beta	Std Error	*t*-Statistic	*p*-Value	Decision
FSK → FSA	0.435	0.034	12.124	<0.001	Supported
FSA → TC	0.037	0.039	0.971	0.332	Not Supported
FSA → personal hygiene	0.247	0.036	2.399	0.017	Supported
FSA → CC	0.048	0.042	1.089	0.277	Not Supported
FSA → HRF	0.169	0.039	2.724	0.007	Supported
Age → FSA	0.04	0.035	1.108	0.268	-
Age → TC	0.226	0.035	6.256	<0.001	-
Age → personal_hygiene	0.139	0.038	3.577	<0.001	-
Age → CC	0.103	0.036	2.809	0.005	-
Age → HRF	0.130	0.038	3.456	0.001	-
Education level → FSA	0	0.037	0.046	0.963	-
Education level → TC	0.175	0.039	4.447	<0.001	-
Education level → personal_hygiene	0.121	0.043	2.776	0.006	-
Education level → CC	0.043	0.041	1.134	0.257	-
Education level → HRF	0.095	0.04	2.345	0.019	-
HCP information access → FSA	0.146	0.041	3.568	<0.001	-
HCP information access → TC	0.123	0.039	3.157	0.002	-
HCP information access → personal_hygiene	0.118	0.04	2.380	0.004	-
HCP information access → CC	0.018	0.04	0.38	0.704	-
HCP information access → HRF	0.051	0.042	1.266	0.206	-

Note: A dash (-) in the Decision column indicates that the relationship is for a control variable and not a direct hypothesis being tested.

**Table 13:** Mediation relationships hypothesis testing among Chinese and Syrian pregnant women.

	Total Effect β	Direct Effect β	Indirect Effects					
			Hypothesis	β	SD	T-Value	Decision	Proportion Mediated (%)
Among Chinese pregnant women								
H3a	0.177 **	0.065	FSK → FSA → TC	0.121 **	0.015	6.089	Supported	68.3
H3b	0.354 **	0.241 *	FSK → FSA → personal hygiene	0.113 *	0.016	4.012	Supported	31.9
H3c	0.436 **	0.278 **	FSK → FSA → CC	0.158 **	0.032	5.818	Supported	36.2
H3d	0.253 **	0.229 *	FSK → FSA → HRFs avoidance	0.024	0.019	1.146	Not Supported	-
Among Syrian pregnant women								
H3a	0.076	0.059	FSK → FSA → TC	0.017	0.021	0.768	Not Supported	-
H3b	0.315 **	0.239 **	FSK → FSA → personal hygiene	0.076 *	0.015	2.098	Supported	24.1
H3c	0.193 **	0.151 *	FSK → FSA → CC	0.042	0.019	1.058	Not Supported	-
H3d	0.293 **	0.201 *	FSK → FSA → HRFs avoidance	0.092 *	0.016	3.463	Supported	31.3

Note: A dash (-) in the Proportion Mediated column indicates that the value is not applicable as the indirect effect was not statistically significant. ** *p* < 0.001, * *p* < 0.05.

**Table 14:** Summary of key cross-cultural differences in food safety KAPs.

KAP Component	Chinese Pregnant Women	Syrian Pregnant Women
FSK	Higher	Lower
FSA	More Positive	Less Positive
FSP	Higher in CC and HRF avoidance	Higher in Personal Hygiene
Direct Effects		
FSK → FSA (H1)	Supported	Supported
FSA → TC Practices (H2a)	Supported	Not Supported
FSA → Personal Hygiene (H2b)	Supported	Supported
FSA → CC Prevention (H2c)	Supported	Not Supported
FSA → HRF Avoidance (H2d)	Not Supported	Supported
Mediating Role of FSA		
FSK → FSA → TC (H3a)	Full Mediation	No Mediation
FSK → FSA → PH (H3b)	Partial Mediation	Partial Mediation
FSK → FSA → CC (H3c)	Partial Mediation	No Mediation
FSK → FSA → HRF (H3d)	No Mediation	Partial Mediation

## Data Availability

The data presented in this study are available on request from the corresponding author. The data are not publicly available due to privacy restrictions.

## Supplementary Materials

The following supporting information can be downloaded at: https://www.mdpi.com/article/10.3390/foods14203564/s1, Table S1: Content validity assessment of the Chinese version and the Arabic version (Syrian version) of the questionnaire item; Table S2: Test-Retest Reliability for the Chinese and Syrian Versions of the Food Safety Questionnaire; Table S3: Assessment Questionnaire of Pregnant Women’s Awareness of Food Safety.

## Abbreviations

The following abbreviations are used in this manuscript:

FBPs	Foodborne pathogens
FBDs	Foodborne diseases
KAPs	Knowledge, attitudes, and practices
FSK	Food safety knowledge
FSA	Food safety attitude
FSP	Food safety practice
CC	Cross-contamination
TC	Temperature control
HCPs	Healthcare providers
PLS-SEM	Partial Least Squares Structural Equation Modeling
HRF	High-risk food
TPB	Theory of planned behavior
S-CVI/Ave	Scale Content Validity Index/Average
CMB	Common method bias
AVE	Average Variance Extracted
CR	Composite reliability
HTMT	Heterotrait–monotrait
