# Supplementary material for: Bridging the Knowledge–Practice Gap: The Culturally Mediated Role of Attitude in Food Safety Behaviors During Pregnancy
Source: Foods. 2025 Oct 20;14(20):3564. doi: 10.3390/foods14203564 (PMC12562517; doi:10.3390/foods14203564)
Supplement: Supplementary file 1 [file foods-14-03564-s001.zip › Table S1.pdf]

Table S1 Content validity assessment of the Chinese version and the Arabic version (Syrian version) of the questionnaire items

Section	Item	Chinese version			Syrian version		
		Experts Rated 3 or 4	I-CVI	S-CVI	Experts Rated 3 or 4	I-CVI	S-CVI
**1. Sources of Food Safety Information**	1	5	1.00	0.90	4	0.80	0.95
	2	4	0.80		5	1.00	
	3	5	1.00		5	1.00	
	4	4	0.80		5	1.00	
**2. Food Safety Knowledge**	1	5	1.00	0.90	5	1.00	0.95
	2	4	0.80		5	1.00	
	3	5	1.00		4	0.80	
	4	4	0.80		5	1.00	
	5	5	1.00		5	1.00	
	6	4	0.80		4	0.80	
	7	5	1.00		5	1.00	
	8	4	0.80		5	1.00	
**3. Food Safety Practice**				0.90			0.94
Cross-Contamination	1	4	0.80	0.93	5	1.00	0.93
	2	5	1.00		5	1.00	
	3	5	1.00		4	0.80	
Temperature Control	1	5	1.00	0.90	4	0.80	0.95
	2	4	0.80		5	1.00	
	3	5	1.00		5	1.00	
	4	4	0.80		5	1.00	
Personal Hygiene	1	5	1.00	0.90	5	1.00	0.95
	2	4	0.80		5	1.00	
	3	5	1.00		4	0.80	
	4	4	0.80		5	1.00	
Consumption of HRFs	1	5	1.00	0.88	4	0.80	0.92
	2	4	0.80		5	1.00	
	3	5	1.00		5	1.00	
	4	4	0.80		5	1.00	
	5	4	0.80		4	0.80	
**4. Food Safety Attitudes**	1	5	1.00	0.92	5	1.00	1.00
	2	5	1.00		5	1.00	
	3	4	0.80		5	1.00	
	4	5	1.00		5	1.00	
	5	4	0.80		5	1.00	
**Overall S-CVI**				0.91			0.96
