# Supplementary material for: Bridging the Knowledge–Practice Gap: The Culturally Mediated Role of Attitude in Food Safety Behaviors During Pregnancy
Source: Foods. 2025 Oct 20;14(20):3564. doi: 10.3390/foods14203564 (PMC12562517; doi:10.3390/foods14203564)
Supplement: Supplementary file 1 [file foods-14-03564-s001.zip › Table S2.pdf]

Table S2 Test-Retest Reliability for the Chinese and Syrian Versions of the Food Safety Questionnaire

	Chinese version			Syrian version		
	Test<br>Mean ±SD	Re-test<br>Mean ±SD	*κ* or *r*	Test<br>Mean ±SD	Re-test<br>Mean ±SD	*κ* or *r*
**Sources Food Safety Information**						
Amount of information	-	-	0.83**	-	-	0.85**
Main source	-	-	0.88**	-	-	0.91**
Must trustworthy source	-	-	0.89**	-	-	0.93**
**Food Safety Knowledge**						
FSK1	3.67±1.0	3.70±1.26	0.84**	3.17±1.20	3.07±1.20	0.89**
FSK2	3.23±1.13	3.60±1.16	0.88**	2.43±0.93	2.83±1.17	0.85**
FSK3	2.77±1.35	3.23±1.04	0.79**	2.47±1.25	3.07±1.01	0.87**
FSK4	3.80±0.92	4.03±0.80	0.82**	3.37±1.12	3.60±0.96	0.84**
FSK5	3.60±0.89	3.63±0.89	0.83**	3.60±1.07	3.83±0.98	0.86**
FSK6	3.50±1.13	3.57±0.89	0.86**	3.27±.98	3.43±0.97	0.81**
FSK7	3.57±0.81	3.73±0.69	0.88**	3.00±1.05	3.33±1.03	0.89**
FSK8	3.93±0.69	4.20±0.61	0.74**	3.63±0.96	3.73±0.82	0.82**
**Cross-Contamination**						
CC1	3.69±1.02	3.74±1.06	0.85**	3.94±1.21	3.99±1.36	0.86**
CC2	3.68±1.04	3.75±1.10	0.84**	3.96±0.99	3.91±1.02	0.83**
CC3	3.62±1.12	3.66±1.14	0.82**	3.68±1.07	3.74±1.13	0.81**
**Temperature Control**						
TC1	3.77±0.93	3.80±0.84	0.81**	3.47±1.04	3.51±0.92	0.83**
TC2	4.00±0.69	4.07±0.69	0.80**	3.73±0.90	3.63±0.76	0.84**
TC3	3.50±0.86	3.33±0.92	0.82**	3.50±0.77	3.47±0.77	0.80**
TC4	3.53±0.81	3.50±0.82	0.82**	3.40±0.96	3.53±0.86	0.81**
**Personal Hygiene**						
PH1	3.93±1.05	4.03±0.81	0.80	3.90±0.87	4.03±1.55	0.84**
PH 2	3.90±0.92	3.97±1.15	0.84	3.97±0.90	4.00±0.95	0.80**
PH 3	4.07±1.21	4.10±0.99	0.81	3.93±1.40	4.00±1.38	0.82**
PH 4	3.87±0.97	3.93±1.36	0.80	3.93±1.21	4.12±1.22	0.83**
**Consumption of HRFs**						
HRF1	4.1±0.91	4.02±1.02	0.82**	4.05±0.89	4.03±1.02	0.86**
HRF 2	3.91±0.96	4.01±0.94	0.85**	4.01±1.05	3.97±1.1	0.83**
HRF 3	3.71±1.01	3.79±1.06	0.83**	3.55±1.01	3.58±0.98	0.88**
HRF 4	3.92±0.97	3.84±0.94	0.82**	3.69±0.91	3.76±1.06	0.81**
HRF 5	3.98±1.07	3.91±1.04	0.84**	3.58±0.93	3.34±1.1	0.87**
**Food Safety Attitudes**						
FSA1	4.05±0.91	4.12±1.03	0.81**	3.85±1.07	3.92±1.05	0.85**
FSA2	4.2±1.03	4.25±0.99	0.88**	3.82±0.95	3.89±0.91	0.88**
FSA3	3.9±0.92	3.83±0.89	0.84**	3.74±0.92	3.81±1.02	0.85**
FSA4	3.75±0.86	3.91±1.06	0.82**	3.53±1.04	3.63±1.06	0.83**
FSA5	3.76±1.01	3.61±0.91	0.80**	3.58±1.02	3.61±1.04	0.89**

Note:
- *κ* (Kappa): Cohen's Kappa, a measure of agreement for categorical items.
- *r:* Pearson's correlation coefficient, a measure of linear correlation for continuous items.
- ** indicates $p < 0.01$.
