# Supplementary material for: Bridging the Knowledge–Practice Gap: The Culturally Mediated Role of Attitude in Food Safety Behaviors During Pregnancy
Source: Foods. 2025 Oct 20;14(20):3564. doi: 10.3390/foods14203564 (PMC12562517; doi:10.3390/foods14203564)
Supplement: Supplementary file 1 [file foods-14-03564-s001.zip › Table S3.pdf]

Table S3 Assessment Questionnaire of Pregnant Women's Awareness of Food Safety

Dear Pregnant Woman.

I am conducting research titled ***"Evaluation the awareness of food safety among pregnant women".***

The attached questionnaire has been designed to gather relevant data for academic research. All responses will remain strictly confidential and will be used exclusively for the objectives of this study.

Kindly answer each question as honestly as possible. Your participation will be highly appreciated.

<u>Completion Guidelines:</u>

This study aims to assess the awareness of food safety among pregnant women.

Please do not write any personally identifiable information in this questionnaire. For each question, mark the checkbox (□) that best reflects your response.

Thank you for participating in this research.

Evaluating Pregnant Women's Awareness of Food Safety

Socio-demographic characterization of the participants

1. How old are you?	□18-24 □ 25-30 □ 31-35 □36-40
2. What is your educational level?	□ Secondary school □ High school □ Graduated □ Post graduated
3. Is this your first pregnancy?	□Yes □No
4. Did you plan for this pregnancy?	□Yes □No
5. How many children do you have?	□ I do not have yet □ 1 □ 2-3 □ more than 3
6. Do you have a history of abortion?	□Yes □No
7. Do you have a job currently?	□Yes □No

Sources of food safety information received during pregnancy

8. How would you describe the amount of information you received about food safety during pregnancy?	□None □Limited □Sufficient □Plenty
9. What is the main source where you get information about food safety during pregnancy?	□ Doctor □ Nutritionist □ Nurse □ TV programs □ Internet □University □ School □ Other □ I do not get any food safety information
10. Which of the following do you consider the most trustworthy source for getting information about food safety during pregnancy?	□ Doctor □ Nutritionist □ Nurse □ TV programs □ Intern □University □ School □ Other
11. Have you got information from most trustworthy source	□Yes □No

Pregnant women's Knowledge of food safety

Please indicate your agreement with the following statements	
12. Salmonella bacteria can cause foodborne illness.	□ Strongly Disagree □ Disagree □ Not Sure □ Agree □ Strongly Agree
13. Toxoplasma gondii can cause foodborne illness.	□ Strongly Disagree □ Disagree □ Not Sure □ Agree □ Strongly Agree
14. Listeria bacteria can cause severe foodborne illness in pregnant women.	□ Strongly Disagree □ Disagree □ Not Sure □ Agree □ Strongly Agree
15. The duration of hand washing is at least 20s with soap before thorough rinsing.	□ Strongly Disagree □ Disagree □ Not Sure □ Agree □ Strongly Agree
16. Cooked food or leftovers that have been left at room temperature for more than 2 hours should not be consumed.	□ Strongly Disagree □ Disagree □ Not Sure □ Agree □ Strongly Agree
17. Tasting is the most reliable way to check if the cooking is sufficient.	□ Strongly Disagree □ Disagree □ Not Sure □ Agree □ Strongly Agree
18. The best way to thaw frozen food is in the refrigerator.	□ Strongly Disagree □ Disagree □ Not Sure □ Agree □ Strongly Agree
19. Raw food and cooked food should be stored separately.	□ Strongly Disagree □ Disagree □ Not Sure □ Agree □ Strongly Agree

Pregnant women's attitude toward food safety

Please indicate your agreement with

20. You are interested in receiving information about food safety during pregnancy	□ Strongly Disagree □ Disagree □ Neutral □ Agree □ Strongly Agree
21. You have paid more attention to food safety after the COVID-19 pandemic	□ Strongly Disagree □ Disagree □ Neutral □ Agree □ Strongly Agree
22. your food safety behaviors have improved after the COVID-19 pandemic	□ Strongly Disagree □ Disagree □ Neutral □ Agree □ Strongly Agree
23. you believe that having proper knowledge about food safety is important for the health of both you and your baby	□ Strongly Disagree □ Disagree □ Neutral □ Agree □ Strongly Agree
24. You are confident in your ability to make safe food choices during pregnancy	□ Strongly Disagree □ Disagree □ Neutral □ Agree □ Strongly Agree

Food safety practices and behaviors among pregnant women.

25. Do you make sure to refrigerate cooked food or leftovers within two hours of cooking?	□Always □Often □Sometimes □Rarely □Never
26. Do you thoroughly reheat cooked foods or leftovers to boiling before eating	□Always □Often □Sometimes □Rarely □Never
27. Do you make sure that your freezer is kept at or below -18°C (0°F)?	□Always □Often □Sometimes □Rarely □Never
28. Do you Make sure that ready to eat food is steaming hot before eating?	□Always □Often □Sometimes □Rarely □Never
29. Do you wash your hands with warm water and soap before eating?	□Always □Often □Sometimes □Rarely □Never
30. Do you wash your hands with warm water and soap before handling the food?	□Always □Often □Sometimes □Rarely □Never
31. Do you wash your hands with warm water and soap after touching raw meat, fish or chicken?	□Always □Often □Sometimes □Rarely □Never
32. Do you wash your hands with warm water and soap after touching raw egg?	□Always □Often □Sometimes □Rarely □Never
33. Do you rinse cutting boards, knives and plates used for raw chicken or meat with hot water and soap before using them for other food?	□Always □Often □Sometimes □Rarely □Never
34. Do you store raw food and cooked food separately?	□Always □Often □Sometimes □Rarely □Never
35. Do you use different chopstick or spoon for uncooked ingredients (meat, poultry, or seafood) and cooked ingredients.	□Always □Often □Sometimes □Rarely □Never
Please read the following foods and choose the option that best reflects your consumption during pregnancy	
36. Pre-prepared salad or Pre-cut fruits	□Always □Often □Sometimes □Rarely □Never
37. Unwashed raw fruits or vegetables.	□Always □Often □Sometimes □Rarely □Never
38. Ready to eat meat served without steaming hot	□Always □Often □Sometimes □Rarely □Never
39. Undercooked meat or foods containing raw meat (e.g., hotpot, barbeque, raw kibbeh)	□Always □Often □Sometimes □Rarely □Never

40. Raw or undercooked eggs, pre-prepared (boiled or fried) egg stored at room temperature	□Always □Often □Sometimes □Rarely □Never
